# Alan Magill, ASTMH Immediate Past President, 1953–2015

**DOI:** 10.4269/ajtmh.935obit

**Published:** 2015-11-04

**Authors:** N. Regina Rabinovich

**Affiliations:** E-mail: rrabinov@hsph.harvard.edu

The sudden and untimely death of Alan Jon Magill at age 61 on September 19, 2015 has led to a growing list of remembrances from a grieving community of friends and colleagues seeking to support his family and has been accompanied by a growing recognition of his extraordinary and unique contributions to the field of tropical diseases. At his death, Alan was the director of the Malaria Program at the Bill & Melinda Gates Foundation (BMGF) since 2012, and was also the immediate past president of the American Society of Tropical Medicine and Hygiene (ASTMH). Both positions underscored his enormous commitment and contributions to tropical medicine over the past decades.

Alan's education was at Lamar University (BS 1976), University of Rhode Island (MS 1978), and Baylor College of Medicine (MD 1984). His post graduate training in the U.S. Army (at Tripler Army Medical Center in Hawaii and at Walter Reed Army Medical Center) was serendipitous, as it allowed him to join his partner and later wife Janiine as she trained as a pediatrician. Thus began the bulk of his career as a physician scientist within the Walter Reed system, combining clinical parasitology and research on product development of new interventions, including drugs and diagnostics.

During his fellowship, Alan first focused on leishmaniasis, as troops from Desert Storm brought home challenging clinical presentations of cutaneous disease. He worked on rapid diagnostic tests and polymerase chain reaction-based assays for cutaneous leishmaniasis; the latter cleared Food and Drug Administration approval. This work established him as a lifelong leishmaniac, and his expertise was firmly grounded in clinical management and his strategic thinking about the need for new interventions. He was subsequently an advisor to leishmania vaccine development programs at the Infectious Diseases Research Institute and the BMGF.

Alan then took on several broad roles; first, on detail to the parasitology program at the U.S. Naval Medical Research Unit No. 6 Peru; second, Chair of the U.S. Army Malaria Board for the Office of the Surgeon General; third, Director of the Division of Experimental Therapeutics at the Walter Reed Army Institute of Research; and finally research coordinator of both the Malaria Drug and Anti-Parasitic Research Programs at the U.S. Army Medical Research and Materiel Command. The breadth of these activities solidified Alan's knowledge of the clinical and product development challenges facing the malaria field.

Alan's achievements in malaria within the Walter Reed Army Institute of Research program included the development of the first American malaria rapid diagnostic test and driving the development of two key treatments, intravenous artesunate and tafenoquine. He identified new malaria drug scaffolds that advanced into lead optimization. He led challenge trials of vaccine candidates in the United States and vaccine field trials in Africa. In addition, while in Peru, he helped to characterize malaria drug resistance patterns at a time when older therapies were losing efficacy, leading to engagement on methods of detection, monitoring of drug resistance, implementation of changes in national policy, and training on the use of new drug regimens.

Subsequently, Alan joined the Defense Advanced Research Projects Agency in 2009, where he directed programs of innovative plant-protein therapeutics and biologics, as well as advancing influenza vaccine candidates into early clinical trials.

Alan's diverse experiences served the malaria field well in 2010, when he participated in the Malaria Eradication Research Agenda (malERA) project. His contributions were critical to the establishment of Single Encounter Radical Cure and Prophylaxis (SERCAP) as a research priority for malaria drug development, enabling progress toward malaria eradication. His contributions as a member of this highly consultative process demonstrated leadership skills, which were to serve him well in his subsequent position at the BMGF.

A listing of Alan's many management and leadership positions almost gets in the way of recognizing his enormous commitment to graduate medical education. His generosity with his time and energy placed him in a vortex of activities to advance graduate medical education of parasitology writ large, and malaria specifically. He was first recognized as an outstanding teaching resident during his internal medicine residency. This was the harbinger for what would become a life-long commitment to education, working through ASTMH, its subgroup, the American Committee on Clinical Tropical Medicine and Travelers' Health (Clinical Group) and the annual Update Course in Clinical and Tropical Medicine, as well as numerous other organizations that represent infectious diseases (Infectious Disease Society of America, American Society of Microbiology) and global health (CDC Traveler's Health and courses sponsored by the Gorgas Course in Tropical Medicine, Universidad Peruana Cayetano Heredia, and Johns Hopkins Bloomberg School of Public Health, to name a few).

Alan's contributions to ASTMH began with his membership in 1991. He served as Councilor, Corporate Liaison, and on the Legislative Affairs Committee. He was a member of the American Committee on Clinical Tropical Medicine and Travelers' Health for 8 years, culminating in his service as President in 2007. He was president of ASTMH in 2014, bringing to this position his remarkable optimism, enthusiasm, and focus. He was seriously engaged in malaria elimination at this time, but during his presidential year a large Ebola outbreak in Africa became a primary focus of the tropical medicine community. He led efforts by the Society to address this epidemic, including careful attention to political challenges, scientific advances and human rights concerns. More broadly, he was greatly interested in the future of the Society, and he left a profound impact on the blueprint for future Society activities.

**Figure:**
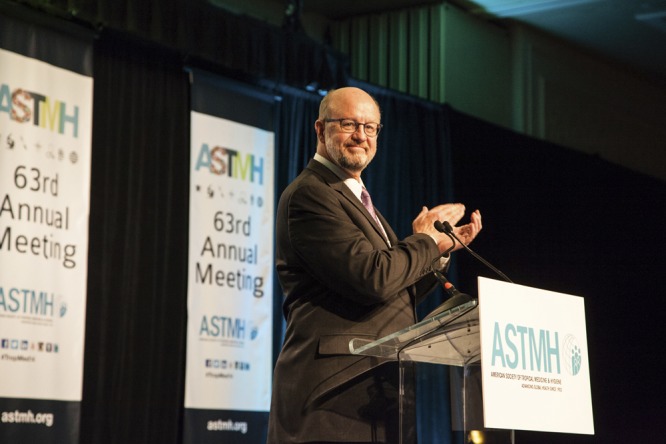
**63rd ASTMH Annual Meeting, New Orleans, Louisiana, November 2, 2014**

Alan's decision to join BMGF as Director of the Malaria Program was not made lightly. He considered the opportunity to drive the Foundation's malaria eradication strategy and its implementation as the culmination of his career. His engaging presentations at the Harvard/Barcelona/Basel Science of Eradication course over the past three years reflected his evolving framework for malaria eradication. Upon arrival at the BMGF, he worked to advance the malaria eradication strategy, applying his strong base as a malaria clinician to rethink the role of drugs as prevention, appropriate strategies to eliminate parasite reservoirs, and reshaping the vaccine development agenda to maximize impact on the eradication of malaria. His strategy was strongly endorsed by Foundation leadership and offered an enormous step forward for the field. Malaria elimination and eventual global eradication are far more achievable goals than we dreamed until very recently, in no small part because of his energy, talent, wisdom, compassion, and commitment.

Through his career, Alan was a master at relating to his team and colleagues. He maintained independence of thought, but was engaged both internally and externally with those that would challenge his thinking. His passion for the history of malaria, understanding of the biological basis of disease, and unique experiences in product development of drugs and vaccines grounded his approach and cemented his credibility with the malaria research and policy communities. The integration of his program into a proactive, forward looking agenda, has resulted in a framework from which it is possible to believe, as he did, that “Malaria Delenda Est”—malaria must be destroyed. This was the inscription on the medal that he awarded, in the military tradition, to the members of his team at the BMGF.

**Figure:**
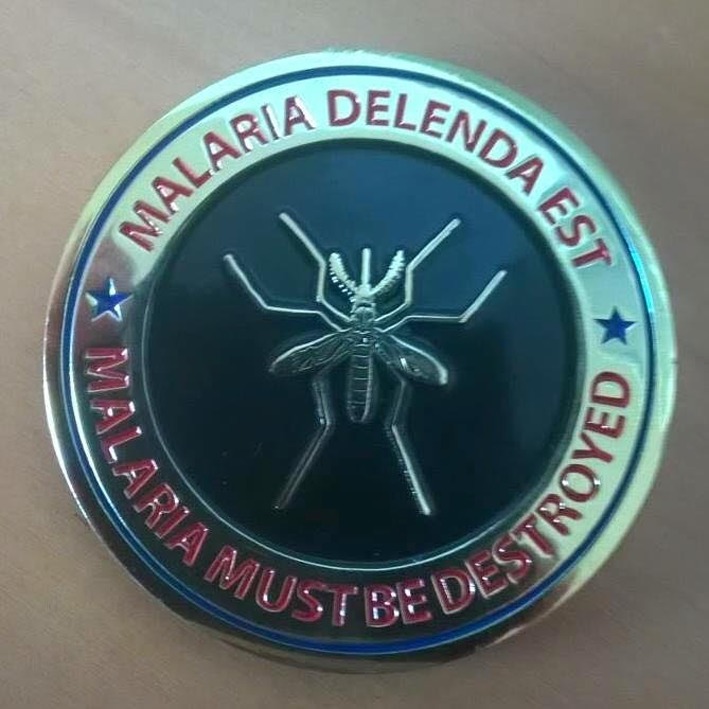
**The medal given by Dr. Magill to his team at the BMGF.**

What is extraordinary is that since Alan's death many people have individually noted how he connected with them personally, giving whatever time was needed. This includes his staff, colleagues, and grantees. Moreover, he managed to do this while also being extraordinarily well-read, a voracious learner, a generous teacher, a malaria expert, a leishmaniac, an historian, a leader, and above all a family man. He certainly came across as smart and hardworking, but he didn't seem extraordinary. As David Brandling-Bennett noted, “After last week, I had to conclude that he was truly extraordinary, but he disguised it so well that individually we didn't realize it.”

An avid outdoorsman, Alan's favorite activities included climbing mountains and hiking, activities which he enjoyed with family and friends, in the Seattle area and beyond. He is survived by his wife Janiine Babcock, daughter Lara Magill and her spouse Jonathan Krynitsky, daughter Sarah Magill, and brother Donald Magill. We thank his family for sharing him with us.

